# Upregulation of the microRNA rno-miR-146b-5p may be involved in the development of intestinal injury through inhibition of Kruppel-like factor 4 in intestinal sepsis

**DOI:** 10.1080/21655979.2020.1851476

**Published:** 2020-11-29

**Authors:** Li Tong, Chaoxia Tang, Changjie Cai, Xiangdong Guan

**Affiliations:** Department of Critical Care Medicine, The First Affiliated Hospital of Sun Yat-sen University, Guangzhou, China

**Keywords:** Intestinal sepsis, RNA sequencing, rno-miR-146b-5p, Kruppel-like factor 4, proliferation

## Abstract

Regulatory mechanisms of microRNAs (miRNAs) in the development of intestinal sepsis are unclear. This study investigated the role of rno-miR-146b-5p in sepsis-induced intestinal injury. A rat sepsis model was created using the cecal ligation and puncture method. The expression profiles of miRNA and mRNA in sepsis rats were examined using miRNA and mRNA sequencing; rno-miR-146b was selected for further investigation. The mimics and inhibitors of rno-miR-146b-5p were transfected into IEC-6 cells and then with or without lipopolysaccharide (LPS) treatment, and the expressions of Kruppel-like factor 4 (Klf4) and Cyclin D2 (Ccnd2) were assessed by quantitative real-time transcriptase-polymerase chain reaction (qRT-PCR) and western blotting. Next, cell counting kit-8 assay was used to detect cell viability, and scratch wound healing assay was used to assess cell migration. In sepsis rat model, crypt cell proliferation was inhibited and crypt cell apoptosis was increased. Compared with the sham control, results of miRNA and mRNA sequencing showed that there were 17 miRNAs and 1617 mRNAs that were upregulated and 123 miRNAs and 1917 mRNAs that were downregulated in the sepsis model group. The network diagrams and qRT-PCR validation indicated that rno-miR-146b-5p may inhibit the expression of Klf4. By adjusting the expression of rno-miR-146b-5p in IEC-6 cells with or without LPS treatment, we found that increased expression of rno-miR-146b-5p inhibited cell proliferation and migration and inhibited the expression of Ccnd2. rno-miR-146b-5p may play a vital role in the development of sepsis intestinal injury through targeting Klf4 expression and affecting promoter activity of Ccnd2.

## 1. Introduction

Sepsis is defined as a life-threatening organ dysfunction caused by a dysregulated host response to infection [1]. Although there have been advances in the prevention and treatment of sepsis, it remains the main cause of mortality in intensive care units [2,3]. The intestinal mucosa acts as a barrier against toxins and bacteria in sepsis. However, during severe sepsis, damage to the intestinal mucosa leads to increased vascular permeability, connection fractures between epithelial cells, edema of the mucosal epithelium, ulcer formation, cell necrosis or apoptosis, enterogenic infection, and intestinal bacterial translocation, resulting in a systemic inflammatory response [4,5]. The intestinal epithelium is continuously replenished from progenitor and stem cells in crypts. Studies have suggested that a crypt base columnar (CBC) stem cell, an actively dividing stem cell, acts as a self-renewing progenitor responsible for maintaining the epithelium [6]. Through stimulation of proliferation and differentiation of intestinal stem cells, CBC stem cells can change the natural course of inflammatory bowel disease, repair damaged intestinal mucosa, and restore the normal function of the intestine. However, the specific mechanisms of action of CBC stem cells remain unknown [7]. Therefore, it is essential to investigate the pathogenesis and repair mechanisms of sepsis-induced intestinal injury to prevent, diagnose, and treat this condition.

MicroRNAs (miRNAs) are a series of endogenous non-coding RNAs that are approximately 21 nucleotides in length. miRNAs are involved in the regulation of gene expression, including regulation of pathways involved in cell proliferation, cell migration, and inflammatory responses [8]. miRNAs have recently been recognized as important post-transcriptional regulators. miRNAs represent approximately 1%–2% of the known genes in eukaryotes and negatively regulate gene expression through base-pairing to target mRNAs [9,10]. Recent reports suggest that miRNAs are involved in the development of sepsis. Evidence suggests that miR-21-3p promotes myocardial injury induced by sepsis [11] and miR-155 alleviates injury in septic rats through inhibition of JNK-related inflammatory responses [12]. miR-25 inhibits cardiomyocyte apoptosis induced by hypoxia/re-oxygenation in sepsis [13]. Other studies have also found that miR-25 is downregulated in the patients with sepsis [14,15]. However, there is little evidence related to the tissue-specific role that miRNAs play in sepsis-induced intestinal injury.

To assess the role of miRNAs in sepsis-induced intestinal injury, sepsis was induced in rats. The jejunal tissue structure and serum levels of TNF-α and IL-1β were assessed to confirm sepsis induction. Sepsis led to the inhibition of proliferation and promotion of apoptosis of intestinal crypt cells in the jejunums of sepsis rats, indicating that the ability of repairing the intestinal crypt was inhibited. To assess the differential expression of miRNA and mRNA, RNA sequencing of jejunal tissues was performed. Bioinformatic analysis, quantitative real-time transcriptase-polymerase chain reaction (qRT-PCR), western blotting (WB), cell counting kit-8 (CCK-8), and scratch wound healing assays were performed to assess the importance of miRNAs in the development of sepsis. Our data indicate that rno-miR-146b-5p and Kruppel-like factor 4 (Klf4) may play an important role in intestinal injury in sepsis.

## 2. Materials and methods

### 2.1 Ethics statement

All animal experiments were performed in accordance with the *Guide for the Care and Use of Laboratory Animals* (National Institutes of Health). All experiments and procedures were reviewed and approved by the ethics committee of the First Affiliated Hospital of Sun Yat-sen University (Guangzhou, China). All efforts were made to minimize the suffering of the animals during experiments.

### 2.2 Cecal ligation and puncture (CLP)-induced sepsis rat model

Adult male Sprague–Dawley (SD) rats (260–300 g) were purchased from Guangdong Medical Experimental Animal Center and randomly categorized into a sham (control) group (n = 5) and sepsis group (n = 5) according to the random number table. CLP-induced sepsis was performed in rats as previously described [16]. In brief, the rats were anesthetized with 4% pentobarbitone (100 μl/100 g body weight) and a 20-mm midline abdominal incision was made to expose the cecum, then ligated just distal to the ileocecal valve to prevent any intestinal obstruction, and punctured twice with an 18-gauge needle. A small quantity of cecal contents was extruded through the puncture wound. The laparotomy incision was closed, and a sterile saline solution (0.9%, 24 mL/kg body weight) was administered for fluid resuscitation. BrdU (50 mg/kg) was intraperitoneally injected 2 h after CLP. At 20 h after CLP, the middle jejunal tissues of the rats were collected; some were flash frozen in liquid nitrogen and some were fixed with 4% paraformaldehyde. Rat serum was also collected and stored at −80°C until further use.

### 2.3 Hematoxylin-eosin (H&E) staining

The formalin-fixed paraffin-embedded jejunal tissue was cut into 4-μm sections and placed on glass slides. Samples were deparaffinated using xylene and washed in ethanol and water. Slides were stained with hematoxylin and washed with water for 10 min followed by an ethanol wash for 5 s. The slides were then stained with eosin and incubated for 10 s. After incubation, slides were washed in water and cover slipped. The images of the slides were obtained by microscopy. Image J software was used to analyze the height of intestinal villi (the vertical distance from the top of villi to the opening of the crypt), the depth of intestinal crypt (the distance from the bottom of crypt to the crypt-villi junction), and the thickness of mucosal layer (the vertical distance from the mucosal epithelium to the mucosal muscle layer).

### 2.4 Enzyme-linked immunosorbent assay (ELISA)

Serum TNF-α and IL-1β levels were measured using commercially available TNF-α (EK0526, Boster Biological Technology co.Itd, Wuhan, China) and IL-1β ELISA kits (EK0393, Boster Biological Technology co.Itd, Wuhan, China) according to the manufacturer’s instructions.

### 2.5 Bromodeoxyuridine immunohistochemistry (BrdU IHC) staining

BrdU is a thymine nucleoside analog that can replace thymine to infiltrate the DNA molecules during cell proliferation, acting as a marker of cell proliferation. Rats were injected with BrdU via intraperitoneal injection and proliferating intestinal crypt cells were visualized by IHC via staining with an anti-BrdU monoclonal antibody (Santa Cruz, sc-51,514).

Intestinal jejunal tissues were treated with formalin for 24 h and paraffinized; 5-μm sections were cut and placed on glass slides, which were then dried at room temperature, dewaxed, hydrated, and treated with 0.1% trypsin in 0.1% calcium chloride at 37°C for 60 min. The slides were treated with 1 M hydrochloric acid at 56°C for 10–40 min. Sections were then stained with a mouse anti-BrdU antibody at a dilution of 1:40 and then visualized using 3,3′-diaminobezidine as the chromogen. Sections were counterstained with hematoxylin for 3 min to visualize unlabeled nuclei.

The proliferation rate of intestinal crypt cells was calculated as a percentage of the number of BrdU positive cells relative to the total number of cells in each crypt and was analyzed using Image J software.

### 2.6 Terminal deoxynucleotidyl transferase UTP nick end labeling (TUNEL) staining

Sections were deparaffinized and washed with PBS followed by TUNEL staining for 60 min at 37°C. Sections were washed twice with PBS and stained with Hoechst solution, followed by three washes with PBS. Stained sections were detected using optical microscopy. Image J software was used to analyze the apoptosis rate in intestinal crypt cells (measured as the ratio of the number of apoptotic cells in each crypt to the total number of cells in each crypt) and apoptosis rate (immunofluorescence positive rate) in jejunal tissues.

### 2.7. miRNA sequencing and mRNA sequencing

Total RNA from the jejunal tissues was purified using the miRNeasy Mini Kit (QIAGEN, Germany). RNA integrity was evaluated based on the RIN value using the Agilent Bioanalyzer 2100 (Agilent, CA, USA). RNA cleanup was performed using the RNA Clean XP Kit (Beckman Coulter, CA, UA), and DNA was removed using the RNase-free DNase Set (QIAGEN, Germany). The quality and concentration of RNA were determined using the NanoDrop 2000 (Thermo Fisher, USA). The miRNA library was prepared using the NEBNext Multiplex Small RNA library (NEB, USA) according to the manufacturer’s instructions. The Agilent Bioanalyzer 2100 was used to evaluate the quality of the library preparation. The Illumina Hiseq 4000 was used for the RNA sequencing, and the subsequent data was assembled and annotated. For the mRNA library, the rRNA was removed using the NEBNext rRNA Depletion Kit (NEB). For library preparation, 1 μg of the total RNA was used according to the manufacturer’s instructions (TrueLib mRNA Library Prep Kit for Illumina, ExCELL, China). RNA was fragmented, double strand cDNA was synthesized, end-polishing was performed, and the cDNA fragments were ligated with adapters. The ligated cDNA was then subjected to universal PCR amplification to obtain sufficient quantities for sequencing. The differentially expressed miRNAs and mRNAs were screened using R software according to fold change in expression (fold change ≥ 1.5) and p-value (p-value < 0.05).

### 2.8 Gene ontology (GO) and Kyoto encyclopedia of genes and genomes (KEGG) pathway analysis

The Database for Annotation, Visualization and Integrated Discovery (http://david.abcc.ncifcrf.gov/) was used to annotate the potential functions in various signaling pathways of the differentially expressed target genes of miRNAs and differentially expressed mRNAs between normal and septic tissues. The genes with a fold difference of ≥1.5 between the normal and septic issues and a p-value of ≤ 0.05 were identified in each dataset. The biological function and pathway of the differentially expressed target genes of miRNAs and differentially expressed mRNAs were enriched in the GO (biological function, cellular component, and molecular function) and KEGG pathway.

### 2.9 miRNA–mRNA network analysis

The network diagram is constructed based on the relationship of the miRNA to its target genes. miRanda (http://www.microrna.org/microrna/home.do) and TargetScan (http://www.targetscan.org/) were used to predict the target genes of differentially expressed miRNAs. The predicted target genes were compared with the mRNA-seq results. Genes with expression patterns that were negatively correlated with their regulatory miRNAs were included. Cytoscape software (Version 3.0; http://www.cytoscape.org/) was used to construct the regulatory network (Shannon et al., 2003). The oval represents miRNA, square represents mRNA, blue indicates significant downregulation, and red indicates significant upregulation.

### 2.10 Cell culture and treatments

In this study, IEC-6 cells were selected to study the function of miR-146b because IEC-6 cells are undifferentiated intestinal epithelial stem cells with proliferative potential and are the precursors of mature epithelial cells. IEC-6 cells are used to study the molecular mechanisms of intestinal mucosal repair and are also commonly used to study the function of intestinal stem cells [17]. IEC-6 cells were obtained from the Cell Culture Center, Chinese Academy of Medical Sciences (Shanghai, China) and were cultured at 37°C with 5% CO_2_. Cells were cultured in Dulbecco’s modified Eagle’s medium with 4 mM L-glutamine, adjusted to contain 1.5 g/L sodium bicarbonate and 4.5 g/L glucose, supplemented with 0.1 units/ml bovine insulin and 10% fetal bovine serum (Gibco, USA). Three days after plating, cells were digested and pelleted by centrifugation. Cell morphology was observed using a light microscope, and cells were suspended at a concentration of 1 × 10^6^/ml. Cells were transfected with miRNA mimics and inhibitors using the RNAiMAX (Thermo Fisher, USA) according to the manufacturer’s instructions. miRNA mimics NC, miRNA mimics, miRNA inhibitors NC, and miRNA inhibitors were purchased from Genepharma (Shanghai, China). The corresponding sequences are as follows: rno-miR-146b-5p mimics sense 5′-UGAGAACUGAAUUCCAUAGGCUGU-3′; rno-miR-146b-5p mimics antisense 5′-AGCCUAUGGAAUUCAGUUCUCAUU-3′ rno-miR-146b-5p mimics NC sense 5′-UUCUCCGAACGUGUCACGUTT-3′; rno-miR-146b-5p mimics NC antisense 5′-ACGUGACACGUUCGGAGAATT-3′; rno-miR-146b-5p inhibitor 5′-ACAGCCUAUGGAAUUCAGUUCUCA-3′; and rno-miR-146b-5p inhibitor NC 5′-CAGUACUUUUGUGUAGUACAA-3′. After transfection for 48 h, IEC-6 cells were treated with 10 ug/mL lipopolysaccharide (LPS) (Sigma, USA) for 24 h and then were collected for further analysis.

### 2.11 CCK-8 assay for cell viability detection

Cells were suspended in 96-well plates at a density of 4 × 10^3^ cells/well. The cells were incubated at 37°C in a 5% CO_2_ humidified incubator. And each well was filled with culture medium to obtain final volume of 100 μl. After incubation for 24, 48 and 72 h, 10 μl of 5 mg/ml CCK-8 (Dojindo, Japan) was added to each well. The cells were incubated with the solution for 4 h. Absorbance was measured at 450 nm using a luminometer (BioTek uQuant, USA).

### 2.12 Wound healing assay

Cells were seeded into 12-well plates, cultured to confluence, and wounded by scraping with a 200-μl pipette tip. The cells were washed by PBS and supplemented with media for 48 h. Images of the cells were then captured using a camera-equipped microscope (Carl Zeiss, NY, USA) at 0, 24, 32, and 48 h after scratching. Image J software was used to calculate the difference of migration area between groups.

### 2.13 qRT-PCR

RNA from jejunal tissues and IEC-6 cells were extracted using the TRIzol reagent (Thermo Fisher Scientific) and miRNeasy Mini Kit (QIAGEN). and reversed-transcribed with a reverse transcription system (Toyobo, Japan). The miRNA and mRNA levels were verified using qRT-PCR. PCR reactions were prepared using the GoTaq qPCR Master Mix (Promega, USA) and were performed using an ABI 7500 system (Applied Biosystem, USA). U6 and GAPDH was used as housekeeping genes to perform comparative quantifications. Primer sequences are presented in Table 1.

**Table 1.** Primer sequences

Primer Name	Sequence (5′-3′)
rno-miR-144-3p-RTprimer(specific)	GTCGTATCCAGTGCAGGGTCCGAGGTATTCGCACTGGATACGACAGTACA
rno-miR-144-3p-F	GCGTACAGTATAGATGATG
rno-miR-146b-5p-RTprimer(specific)	GTCGTATCCAGTGCAGGGTCCGAGGTATTCGCACTGGATACGACACAGCC
rno-miR-146b-5p-F	AACACGCTGAGAACTGAATTCC
rno-miR-383-5p-RTprimer(specific)	GTCGTATCCAGTGCAGGGTCCGAGGTATTCGCACTGGATACGACCCACAG
rno-miR-383-5p-F	AACACGCCAGATCAGAAGGT
miRNA universal reverse primer	GTGCAGGGTCCGAGGT
Rat-Tlr2-F	TGGAGGTCTCCAGGTCAAATC
Rat-Tlr2-R	TGTTTGCTGTGAGTCCCGAG
Rat-Klf4-F	TCACATGAAGCGACTTCCCC
Rat-Klf4-R	CGTTGAACTCCTCGGTCTCC
Rat-Neurod1-F	ACAGACGAGTGCCTCAGTTC
Rat-Neurod1-R	CCCGTCTCTTGGGCTTTTGA
Rat-Gapdh-F	GCAAGAGAGAGGCCCTCAG
Rat-Gapdh-R	TGTGAGGGAGATGCTCAGTG
rno -U6-RT	AACGCTTCACGAATTTGCGT
rno -U6-F	CTCGCTTCGGCAGCACA

### 2.14 WB

Cells were lysed in 1% SDS lysis buffer. BCA assay was performed to determine the concentration of protein. 10% SDS-PAGE was used to separate the protein which was then transferred onto polyvinylidene fluoride (PVDF) membranes. Nonfat milk in PBS was used to block the membrane at room temperature for 1 h. The membrane was incubated overnight at 4°C with Klf4 primary antibody (11880-1-AP, Proteintech, China), Ccnd2 primary antibody (3741, CST, USA), or GAPDH primary antibody (60004-1-1 g, Proteintech). After several washes with TBST, membranes were incubated in blocking buffer with a secondary antibody coupled to horseradish peroxidase for 2 h at room temperature. The blot was imaged using the ECLplus (Amersham Biosciences/GE Healthcare, Velizy, France). The optical density of the target band was analyzed using Image J software. GAPDH was used as an internal reference to compare the expression differences of target proteins after different treatments.

### 2.15 Statistical analysis

When data corresponds to normal distribution, comparisons were performed using *t*-tests or analysis of variance (ANOVA). P-values of ≤ 0.05 were considered statistically significant. GraphPad Prism software was used for statistical analysis.

## 3. Results

### 3.1 Development of the intestinal injury model of sepsis in rats

**Figure 1.**
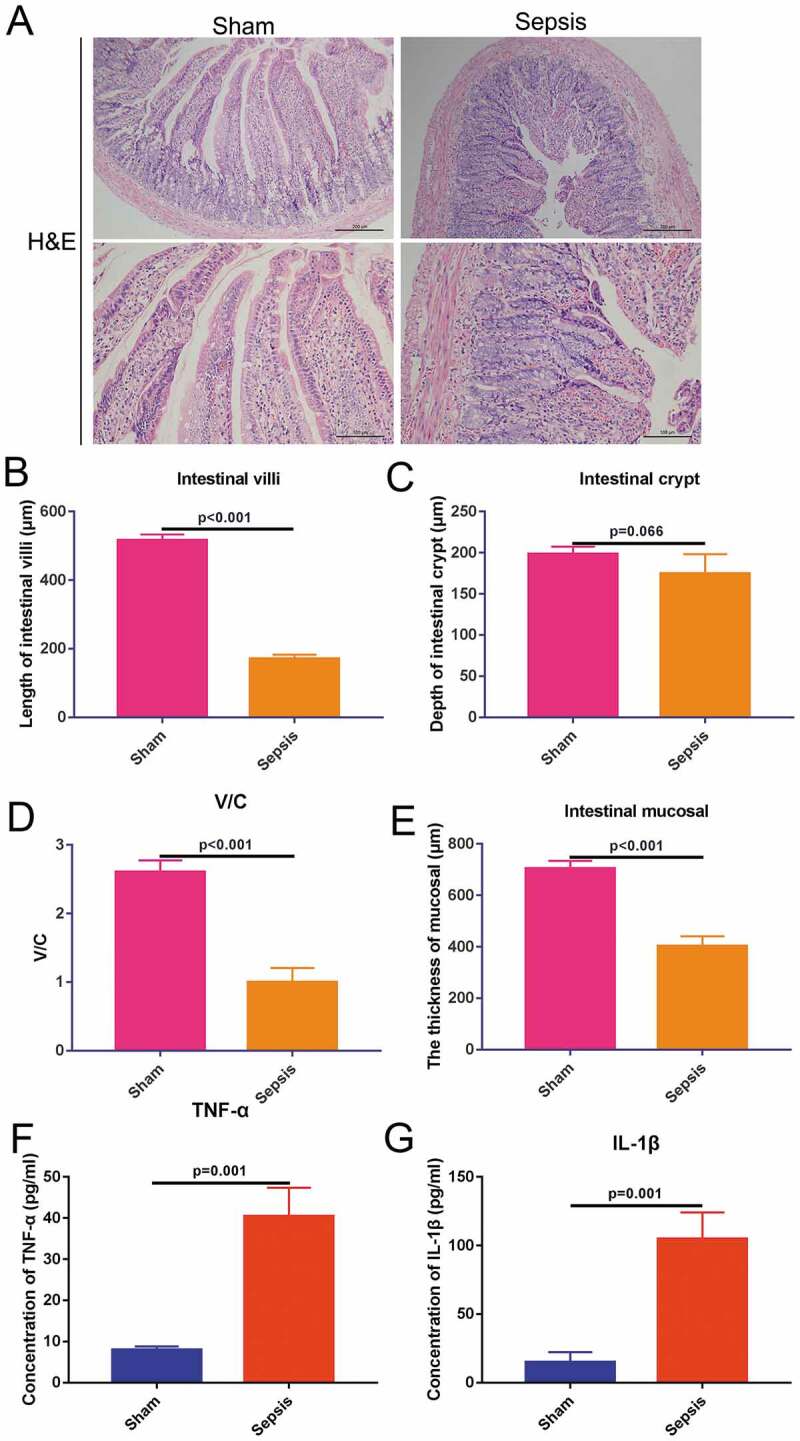
Sepsis rat model validation. (a). Hematoxylin-eosin staining showing jejunal tissue structures in the sham and sepsis groups. Above: 100×, scaled bar: 200 μm; down: 200×, scaled bar: 100 μm; (b). Length of intestinal villi in each group. (c). Depth of intestinal crypt in each group. (d). Ratio of villi and crypt in each group. (e). The thickness of mucosal membrane in each group. (f). Concentration of TNF-α in the serum in each group. (g). Concentration of IL-1β in the serum in each group

The sepsis rat model was induced using the CLP method. At 2 h after sepsis induction, 50 mg/kg BrdU was administered via intraperitoneal injection. Middle jejunal tissues were collected and stained with H&E to validate the sepsis animal model. The jejunal tissues of the sham group exhibited regular cell structure and had clear aligned intestinal villi. However, the jejunal tissues of the sepsis group displayed disordered jejunal structure, and intestinal mucosa was shortened and shed (Figure 1(a)). Compared with the sham group, the length of the intestinal villi was decreased to approximately 40% (Figure 1(b)), although there were no significant alterations in the depth of the intestinal crypt (Figure 1(c)). Previous reports suggest that the ratio of the length of intestinal villi to the depth of intestinal crypt (V/C value) more comprehensively reflects the functional state of the small intestine [18]. Therefore, we analyzed the V/C value. Compared with the sham group, the V/C value of the sepsis group was significantly reduced, indicating a decrease in intestinal digestion and absorption (Figure 1(d)). The thickness of the mucosal layer was also decreased in the sepsis group to approximately 50% of the thickness of the sham group (Figure 1(e)). Serum levels of the inflammatory cytokines TNF-α and IL-1β were significantly increased (5-fold) in the sepsis group (p ≤ 0.05; figure 1(f) & 1(g)). Based on the alterations observed in the jejunal tissue structure and increased levels of inflammatory cytokines in the serum, we concluded that sepsis was successfully induced.

### 3.2. Sepsis inhibits proliferation and promotes apoptosis of intestinal crypt cells in the jejunal tissue

**Figure 2.**
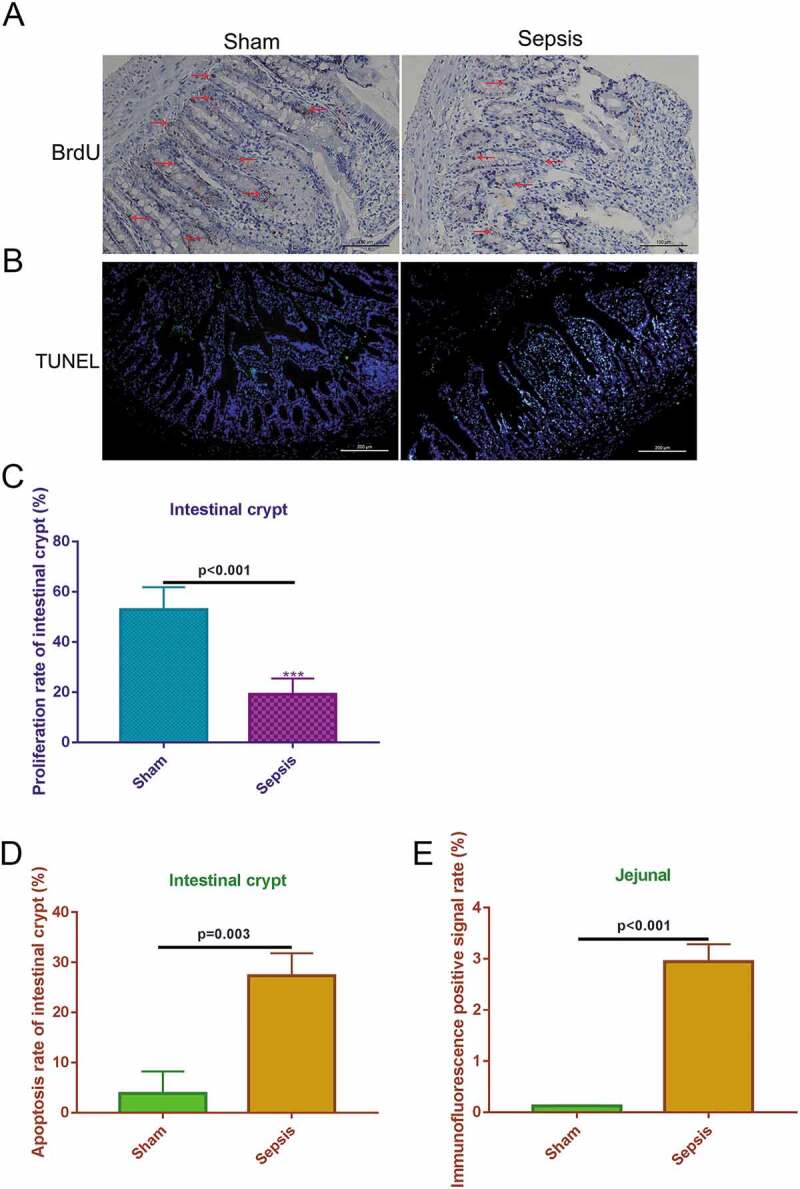
Sepsis suppresses cell proliferation and induces cell apoptosis in intestinal crypt and jejunal tissue. (a). Bromodeoxyuridine immunohistochemistry staining showing cell proliferation of the intestinal crypt in the control and sepsis groups. Magnification: 200 ×. Scale bar: 100 μm. (b). The Terminal Deoxynucleotidyl Transferase UTP Nick End Labeling staining to detect cell apoptosis in the sham and sepsis groups. 100×, scaled bar: 200 μm (c). Cell proliferation rate of intestinal crypt in each group. (d). Cell apoptosis rate of intestinal crypt in each group. E. Immunofluorescence positive signal rate of the jejunum in each group

To measure the proliferation levels in the jejunal tissues, BrdU IHC staining was used to estimate proliferation based on the amount of stained cells in each crypt. The proliferation rate of the crypts in the sepsis group was obviously decreased compared with that in the sham control (Figure 2(a) & 2(c)). To measure the apoptosis levels in the intestinal crypt and jejunum, TUNEL staining was used to stain apoptotic cells. The number of apoptotic cells was greater in the sepsis group than in the sham group (Figure 2(b)). In the sepsis group, approximately 30% of the intestinal crypt cells were TUNEL-positive, whereas in the sham group only approximately 3% of cells were TUNEL-positive (Figure 2(d)). After assessing the entire jejunal tissue, 3% of total cells were TUNEL-positive in the sepsis group, whereas < 1% of cells were TUNEL-positive in the sham group (Figure 2(e)). These results indicate that sepsis suppresses the proliferation of intestinal crypt cells in jejunal tissues and also induces apoptosis.

### 3.3. miRNAs and mRNAs involved in intestinal injury in sepsis

**Figure 3.**
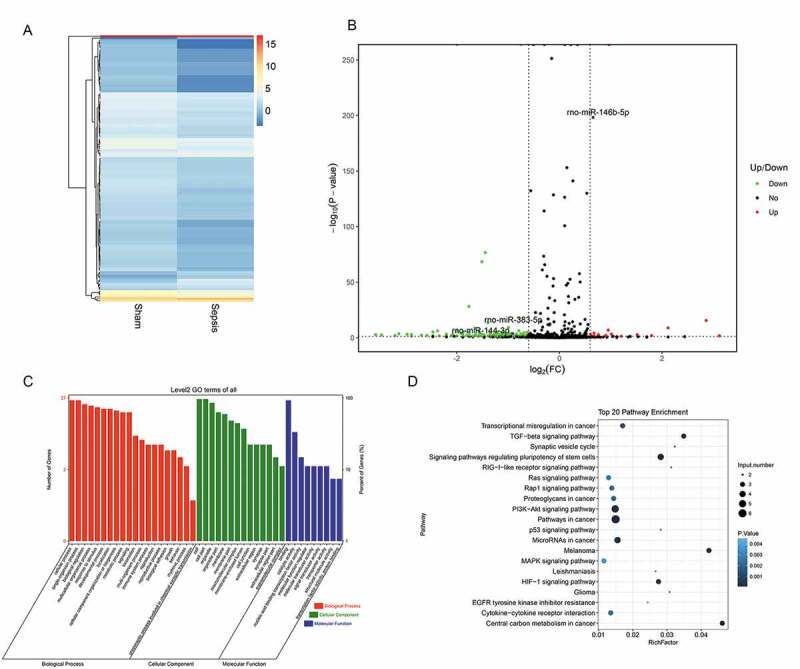
microRNA (miRNA) expression profiles in the jejunum with sepsis. (a). Cluster graph of the differentially expressed miRNAs. (b). Volcano plot of the differentially expressed miRNAs. (c). Gene Ontology analysis to predict target genes of differentially expressed miRNAs. (d). Kyoto Encyclopedia of Genes and Genomes pathway annotations of the predicted target genes of differentially expressed miRNAs

**Figure 4.**
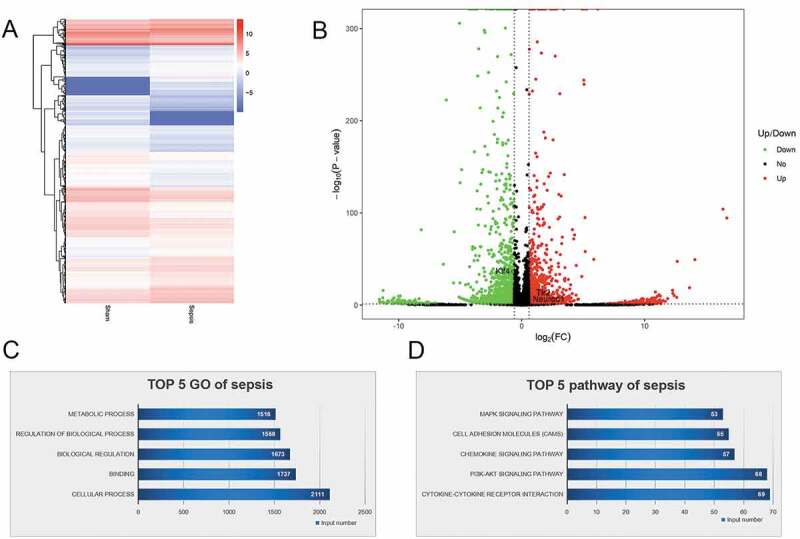
mRNA expression profiles in the jejunum with sepsis. (a). Cluster graph of the differentially expressed mRNA. (b). Volcano plot of the differentially expressed mRNAs. (c). GO analysis of the differentially expressed mRNAs. (d). KEGG pathway annotation of the differentially expressed mRNAs

To identify miRNAs and mRNAs that are involved in intestinal injury in sepsis and elucidate the pathogenic mechanisms of sepsis, we performed RNA sequencing. We assessed differentially expressed miRNAs between the sepsis and sham groups. The findings are summarized in the cluster graph shown in Figure 3(a) and Supplemental Table 1. A volcano plot was generated to assess the differentially expressed miRNAs in sepsis (Figure 3(b)). Compared with the sham group, 140 miRNAs were differentially expressed (fold change ≥ 1.5) in the sepsis group, with 17 miRNAs that were significantly upregulated and 123 miRNAs that were significantly downregulated (Supplemental Table 1). Based on the GO analysis, the target genes of significantly differentially expressed miRNAs were commonly involved in biological processes, cellular components, and molecular function (Figure 3(c)). KEGG pathway analysis found that the target genes of the differentially expressed miRNA were significantly enriched in signaling pathways, including pathways associated with cancer, PI3K-Akt pathway, signaling pathways regulating pluripotency of stem cells, cytokine–cytokine receptor interaction, MAPK signaling pathway, and TGF-beta signaling pathway (Figure 3(d)). In addition to the miRNA levels, the expression of mRNAs was also assessed via RNA sequencing to investigate the interaction between miRNAs and mRNAs. The resulting mRNA expression profile was summarized using a cluster graph (Figure 4(a), Supplemental Table 2). Compared with the sham group, in total, 1,617 mRNAs were upregulated and 1,917 mRNAs were downregulated in the sepsis group; differentially expressed mRNAs were also shown in a volcano plot (Figure 4(b)). GO analysis presented the top five functional processes of the differentially expressed mRNAs, and cellular process was the most enriched process (Figure 4(c)). KEGG pathway annotation for the differentially expressed mRNAs suggests that the cytokine–cytokine receptor interaction and PI3K-Akt pathway were the two most enriched pathways for the differentially expressed mRNAs (Figure 4(d)).

### 3.4 qRT-PCR confirms miRNA and mRNA sequencing data

**Figure 5.**
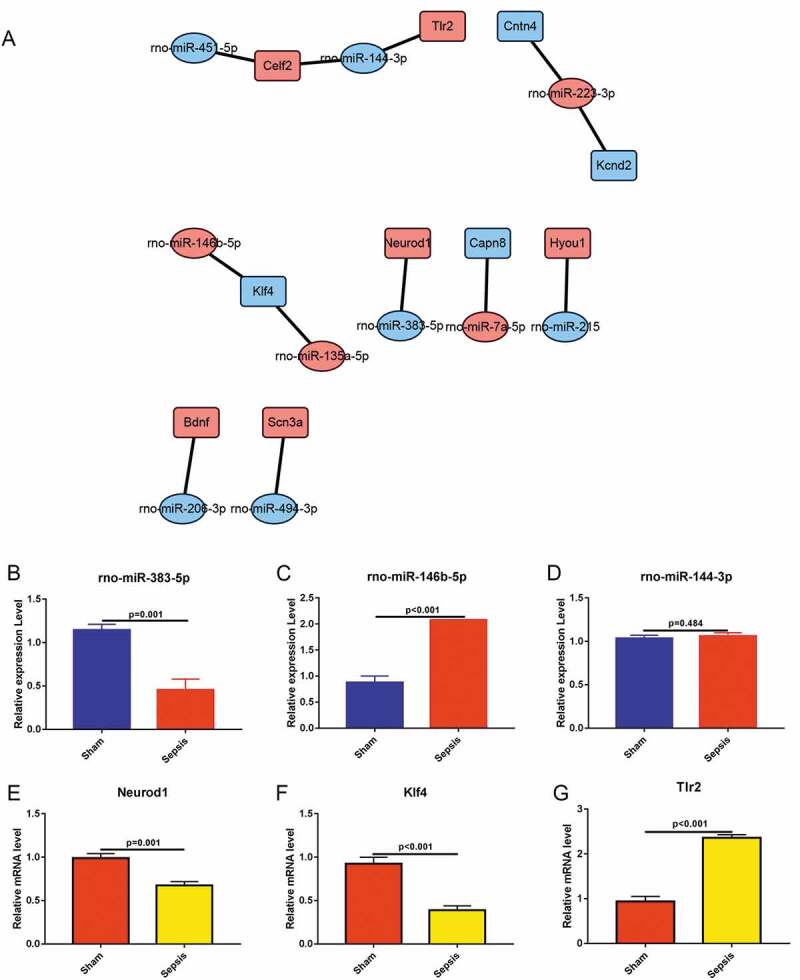
Quantitative reverse transcriptase PCR (qRT-PCR) was conducted to validate the differentially expressed miRNAs and mRNAs. (a). Predictive regulation cascade of the differentially expressed miRNAs and mRNAs. (b). qRT-PCR in the validation of rno-miR-383-5p. (c). qRT-PCR in the validation of rno-miR-146b-5p. (d). qRT-PCR in the validation of rno-miR-144-3p. (e). qRT-PCR in the validation of neuronal differentiation 1. (f). qRT-PCR in the validation of Kruppel-like factor 4. (g). qRT-PCR in the validation of toll-like receptor 2

To confirm the findings of RNA sequencing, we performed miRNA–mRNA network and using qRT-PCR, measured the levels of certain miRNAs and mRNAs. Based on the profile of miRNA and mRNA, we constructed miRNA–mRNA network between the differentially expressed miRNAs and differentially expressed mRNAs (Figure 5(a)). We selected three key mRNAs that were associated with sepsis intestinal injury and identified corresponding miRNAs from the miRNA–mRNA network diagram based on the function of the mRNAs. We used qRT-PCR to detect the expression levels of these miRNAs and mRNAs in the jejunal tissues from both the sham and sepsis groups. qRT-PCR findings showed that rno-miR-383-5p was significantly downregulated and rno-miR-146b-5p was upregulated in the sepsis group (Figure 5(b) & 5(c)), which was consistent with the results of miRNA sequencing (Figure 3(b) and Supplemental Table 1). There were no differences in the expression levels of rno-miR-144-3p between the sepsis group and sham group (Figure 5(d)), which is inconsistent with the results of the miRNA sequencing (Figure 3(b) and Supplemental Table 1). We also measured mRNA levels of Neurod1 and Klf4, which were downregulated in the sepsis group (Figure 5(e) & 5(f)), and levels of Tlr2, which were upregulated in the sepsis group (Figure 5(g)). Among these genes, the expression levels of Neurod1 were not consistent with the findings of the mRNA sequencing (Figure 4(b) and Supplemental Table 2). However, the expression levels of Klf4 and Tlr2 were consistent with the findings of the mRNA sequencing (Figure 4(b) and Supplemental Table 2). As miRNA typically inhibits the expression of its target genes, we hypothesized that rno-miR-146b-5p inhibits Klf4 in the development of sepsis.

### 3.5. Rno-miR-146b-5p may suppress proliferation and migration of IEC-6 cells through inhibition of the expression of Klf4

**Figure 6.**
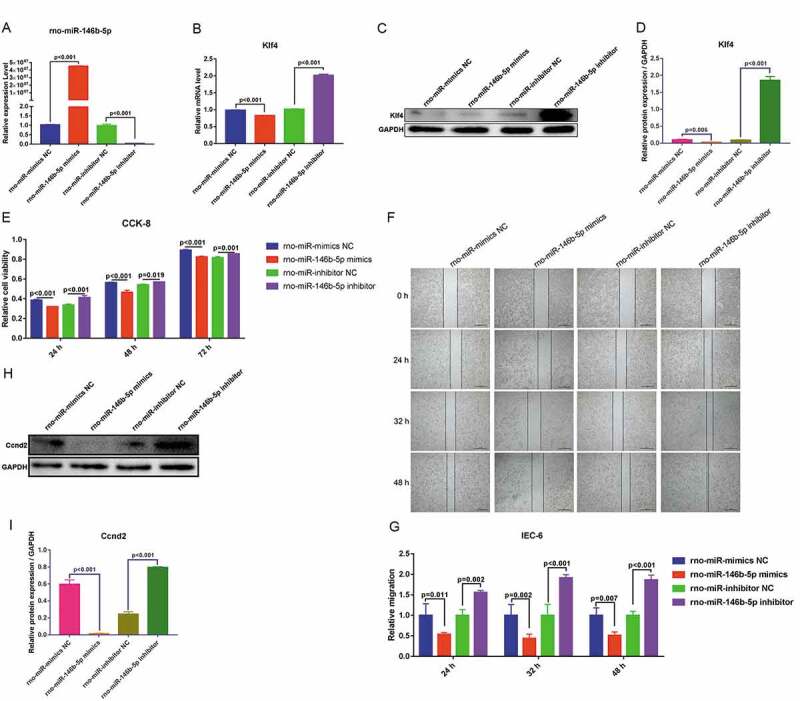
Rno-miR-146b-5p may affect the proliferation and migration of IEC-6 cells by targeting and regulating Klf4 expression and affecting the promoter activity of cyclin D2 (Ccnd2). (a). Rno-miR-146b-5p levels after IEC-6 cells were transfected with mimics and inhibitor. (b). Klf4 mRNA levels after IEC-6 cells were transfected with rno-miR-146b-5p mimics and inhibitor. (c&d). Klf4 protein levels after IEC-6 cells were transfected with rno-miR-146b-5p mimics and inhibitor. (e). Cell counting kit-8 assay in the detection of cell viability in each group. (f). Cell scratch wound healing assay to determine the capacity of cell migration. (g). Relative migration area of the IEC-6 cells in scratch wound healing assay. The results of statistical analysis of three independent replicates are also shown. (h&i). Protein level of Ccnd2 after IEC-6 cells were transfected with rno-miR-146b-5p mimics and inhibitor

To validate the role of rno-miR-146b-5p and Klf4 in regulating cell proliferation and migration, rno-miR-146b-5p-specific mimics and inhibitors were prepared and transfected into IEC-6 cells. The performances of the mimics and inhibitors were determined by qRT-PCR. rno-miR-146b-5p mimics increased overall levels of rno-miR-146b-5p, whereas the inhibitors decreased the levels of rno-miR-146b-5p (Figure 6(a)). The mRNA levels of Klf4 were significantly decreased when the cells were transfected with rno-miR-146b-5p mimics, whereas the levels increased 2-fold when cells were transfected with the rno-miR-146b-5p inhibitor (Figure 6(b)). Treatment with rno-miR-146b-5p mimics decreased the protein levels of Klf4, whereas the corresponding inhibitor increased the protein levels of Klf4 as measured by WB (Figure 6(c) & 6(d)). These results were consistent with the mRNA levels of Klf4. Therefore, the correlation between rno-miR-146b-5p and Klf4 levels was consistent with our hypothesis.

To determine the capacity for cell proliferation, we used a CCK-8 assay. Treatment with rno-miR-146b-5p mimics led to a significant decrease in cell viability at 24, 48, and 72 h after transfections compared with that after treatment with the NC mimetic. The cell viability was significantly increased when cells were transfected with an rno-miR-146b-5p inhibitor for 24, 48, and 72 h as compared with cells treated with an NC inhibitor ((Figure 6e)). To determine cell migration, we performed a cell scratch wound healing assay and found that migration was slower in the cells treated with the rno-miR-146b-5p mimics than in those treated with the NC mimetic. Cells treated with the rno-miR-146b-5p inhibitor migrated more compared with those treated with the corresponding inhibitor NC (figure 6(f)). Quantification of the normalized migration area showed that the migration capacity decreased to approximately 50% in the rno-miR-146b-5p mimics group but increased to approximately 50% in the rno-miR-146b-5p inhibitor group, compared with the corresponding NC group at 24, 32, and 48 h after transfection (Figure 6(g)). These data indicated that the upregulation of rno-miR-146b-5p may inhibit cell proliferation and migration and downregulation of rno-miR-146b-5p may enhance cell proliferation and migration.

To further validate the regulatory mechanisms of rno-miR-146b-5p in IEC-6 cells’ function, we conducted a joint analysis of miRNA and mRNA sequencing data. We found that differentially expressed miRNAs and mRNAs were involved in the PI3K-Akt signaling pathway. mRNA sequencing data showed that Ccnd2, a molecule closely related to the cell cycle in the PI3K-Akt signaling pathway, was significantly downregulated in the sepsis group. To further assess this, we measured protein levels of Ccnd2 and found reduced Ccnd2 protein when rno-miR-146b-5p was increased and increased Ccnd2 protein levels when the expression of rno-miR-146b-5p was suppressed by the inhibitor (Figure 6(h) & 6(i)). Based on these results, we suggest that rno-miR-146b-5p regulates the capacity of cell proliferation and migration through the regulation of the expression of Klf4.

### 3.6. Rno-miR-146b-5p may suppresses lipopolysaccharide-treated IEC-6 cells proliferation and migration by inhibiting Klf4 expression

**Figure 7.**
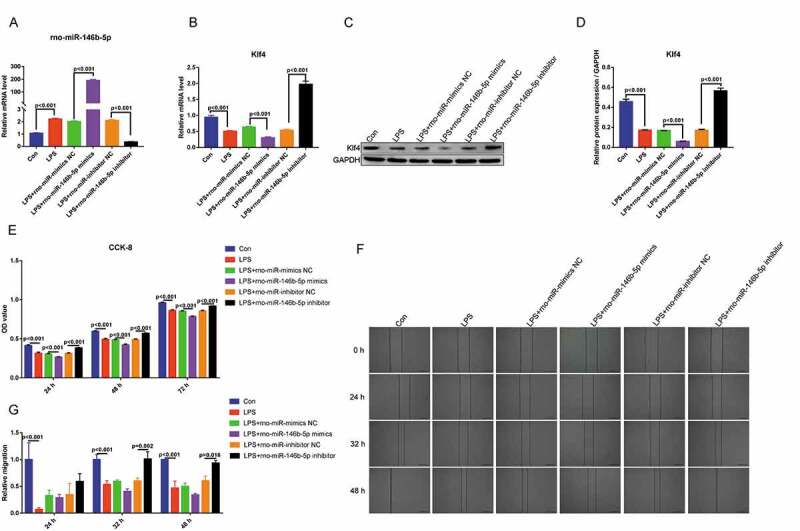
Rno-miR-146b-5p affects the proliferation and migration of LPS-treated IEC-6 cells by regulating Klf4 expression. (a). The level of rno-miR-146b-5p in LPS-treated IEC-6 cells transfected with the mimics or inhibitor of rno-miR-146b-5p. (b). The level of Klf4 mRNA in LPS-treated IEC-6 cells transfected with rno-miR-146b-5p mimics or inhibitor. (c&d). The protein level of Klf4 in LPS-treated IEC-6 cells transfected with rno-miR-146b-5p mimics or inhibitor. (e). The cell viability in each group by the cell counting kit-8 assay. (f). Cell scratch wound healing assay is used to assay cell migration. (g). Relative migration area of the cells in the scratch wound healing assay. The results of statistical analysis of three independent replicates are also shown

To further investigate the effect of rno-miR-146b-5p on in vitro inflammation cell model, IEC-6 cells were transfection with the mimics and inhibitors of rno-miR-146b-5p, and then were treated with LPS. The expression of rno-miR-146b-5p was determined by qRT-PCR. Compared with the control group (Con), rno-miR-146b-5p was significantly upregulated in the LPS group. The rno-miR-146b-5p mimics increased the overall level of rno-miR-146b-5p, whereas the inhibitors decreased the overall level of rno-miR-146b-5p in LPS-treated IEC-6 cells (Figure 7(a)). Compared with the control group, the mRNA and protein levels of Klf4 were significantly decreased in the LPS group and further decreased when transfected with rno-miR-146b-5p mimics and treated with LPS. In contrast, in cells transfected with the rno-miR-146b-5p inhibitor and treated with LPS, the mRNA and protein levels of Klf4 significantly increased when compared with control group(Figure 7(b–d)).

The CCK-8 assay demonstrated that the viability of the LPS-treated IEC-6 cells were significantly decreased at 24, 48, and 72 h. Compared with the control group, transfection with rno-miR-146b-5p mimics and treatment with LPS further decreased the cell viability at 24, 48, and 72 h. Compared with transfected with rno-miR-146b-5p inhibitor and treated with LPS group, the cell viability was significantly increased when transfected with rno-miR-146b-5p inhibitor and treated with LPS at 24, 48, and 72 h (Figure 7(e)). The cell scratch wound healing assay showed that cell migration was slower in the LPS group than in the control group. Cell migration was also inhibited when cells were transfected with rno-miR-146b-5p mimics and treated with LPS compared with transfected with NC mimetic and treated with LPS group; in contrast, the rno-miR-146b-5p inhibitor significantly enhanced the migration under LPS treatment when compared with transfected with inhibitor NC and treated with LPS group (figure 7(f) and (g)). The above results indicate that rno-miR-146b-5p is involved in sepsis-induced intestinal injuries by inhibiting cell proliferation and migration via regulating the expression of Klf4.

## 4. Discussion

Sepsis is defined as a life-threatening organ dysfunction caused by a dysregulated host response to infection [1]. Sepsis can cause intestinal epithelial cell dysfunction, including increased epithelial cell apoptosis, reduced intestinal crypt cell proliferation, barrier dysfunction, and cytokine production [19,20]. In this study, we found disordered jejunal tissue structure in the sepsis model group, with decreased length of intestinal villi, reduced V/C values, reduced thickness of the mucosal layer, and increased levels of serum TNF-α and IL-1β. Meanwhile, intestinal crypt cell proliferation was inhibited and cell apoptosis was increased in the jejunal tissue of the sepsis group. Intestinal mucosal epithelial cells are constantly self-renewing and their function depends on the continuous proliferation and differentiation of crypt stem cells to replace the outer terminally differentiated cells. Reports suggest that stimulating the proliferation and differentiation of crypt stem cells alters the natural course of inflammatory bowel disease, repairs damaged intestinal mucosa, and restores the normal function of the intestine [7]; however, specific mechanisms of action remain poorly understood.

miRNAs are a class of small RNA molecules that participate in and play an important role in the regulation of various cellular processes. Studies have shown that miRNAs play an important role in inflammatory responses and immune regulation in sepsis [21,22]. The present study found that the miRNA and mRNA expression profiles in the jejunal tissues in rats are altered with sepsis. KEGG pathway analysis revealed that the PI3K-Akt pathway is a key signaling pathway altered by these differentially expressed miRNAs and mRNAs and may be involved in sepsis intestinal injury. Gayer et al. found that strain-induced proliferation of IEC-6 cells is achieved through PI3K-Akt signaling pathways [23]. Hsu et al. found that *Klebsiella pneumoniae* translocates across the intestinal epithelium via rho GTPase and PI3K-Akt-dependent cell invasion [24]. Therefore, we propose that the activation of PI3K-Akt signaling pathway plays an important role in the intestinal injury of sepsis.

We found that rno-miR-146b-5p and Klf4 were enriched in the PI3K-Akt signaling pathway, and rno-miR-146b-5p was upregulated in the sepsis rat model. Moreover, the increased expression of rno-miR-146b-5p inhibited cell proliferation and migration, indicating its role in sepsis-induced intestinal injuries. The involvement of miR-146b in inflammation has been the focus of research [25]. Our results are consistent with those of a previous study that demonstrated that miR-146b-5p was upregulated in the myocardium in sepsis [21]. Other evidence suggests that miR-146b-5p also induces cardiomyocyte apoptosis through increasing levels of hypoxia [26]. Another study reported that the overexpression of miR-146b relieved intestinal inflammation by activating the NF-κB pathway in mice with DSS-induced colitis [27]. However, the contradictory reports that hsa-miR-146b is upregulated in sepsis [21] but downregulated in colitis [27] add to the confusion. It is possible that miR-146b’s function varies under different conditions, and therefore needs to be studied further *in vitro* and *in vivo*. In the present study, we hypothesized that rno-miR-146b-5p is involved in intestinal injury during sepsis.

The transcription factor Klf4 was a predicted target of rno-miR-146b-5p and was downregulated during intestinal sepsis. Klf4 belongs to a 17-member family of transcription factors that play a role in regulating gene expression [28,29]. Klf4 is expressed in various cell types and is an epithelial-specific transcription factor involved in regulating cell growth and differentiation [30]. Talmasov et al. found that Klf4 is a radioprotective factor for the intestine after γ-radiation-induced gut injury in mice [31]. In addition, Klf4-deficient mice exhibit intestinal injury and have a significantly increased rate of apoptosis of intestinal crypt cells [31]. In the present study, Klf4 was downregulated in the jejunal tissues of rats with sepsis, and decreased Klf4 expression inhibited the proliferation and promoted the apoptosis of intestinal crypt cells.

Bioinformatic analysis and qRT-PCR validation indicated that rno-miR-146b-5p might regulate Klf4. It was later verified that Klf4 expression was significantly decreased or increased by transfection with rno-miR-146b-5p mimics or rno-miR-146b-5p inhibitor, respectively. These data are consistent with Ge et al.’s report that miR-146b specifically regulates the translation of Kruppel-like factor 4 (KLF4) by targeting the 3′ untranslated region of its mRNA [32]. These data suggest that rno-miR-146b-5p is involved in sepsis-induced intestinal injuries via the inhibition of Klf4.

To determine the signaling pathway regulated by Klf4 which leads to alterations in cell proliferation and migration, we performed bioinformatics analysis of miRNA and mRNA sequencing results. Our findings suggest that the PI3K-Akt pathway plays an important role in intestinal injury in sepsis. To further assess this, we analyzed mRNAs that were differentially expressed and examined the mRNAs that were enriched in the PI3K-Akt signaling pathway. We found that Ccnd2 is an important factor in the regulation of cell proliferation and was significantly downregulated in the sepsis group. Ccnd2 is a cell cycle activator capable of moving the cell cycle from the G1 phase to S phase [33].We measured Ccnd2 in IEC-6 cells after transfection of rno-miR-146b-5p mimics and inhibitors and found that the expression of Ccnd2 was negatively correlated with the expression of rno-miR-146b-5p. Interestingly, bioinformatic analysis found no potential binding sites between Ccnd2 and rno-miR-146b-5p; therefore, we suggested that rno-miR-146b-5p may indirectly regulates Ccnd2 via affecting Klf4. Previous studies suggested that Klf4 knockout mice show significantly reduced expression of Ccnd2 and inhibited proliferation of B cells. Additionally, previous study also found that Klf4 can directly target the promoter region of Ccnd2 to regulate its expression [34]. Therefore, Klf4/Ccnd2 can regulate the G1-to-S phase transition of the cell cycle, affecting the proliferation of B cells [33]. Thus, we suspect that the upregulation of rno-miR-146b-5p in the sepsis model may inhibit the promoter activity of Ccnd2 owing to reduced Klf4 expression. This will lead to decreases in Ccnd2 expression, reduced G1-to-S phase transition of the cell cycle, and ultimately inhibited IEC-6 cell proliferation.

Although this study identifies potential pathogenic mechanisms of sepsis, there are limitations. Further future studies are needed to fully elucidate the relationship between rno-miR-146b-5p and Klf4, determine the functions of Klf4 and rno-miR-146b-5p *in vivo* through overexpression or interference techniques, identify the relationship between Klf4 and Ccnd2, and identify the miR-146b-5p/Klf4/Ccnd2 cascade in the development of sepsis *in vivo*.

## 5. Conclusions

Our results suggested the potential cascade of rno-miR-146b-5p, Klf4, and Ccnd2 in regulating the pathogenic processes underlying sepsis-induced intestinal injury. Rno-miR-146b-5p may be involved in the development sepsis by targeting and regulating the expression of Klf4. Klf4 may inhibit the expression of Ccnd2 by affecting its promoter activity, thereby inhibiting the progression of cell cycle and subsequent cell proliferation. This pathway may be an underlying mechanism of intestinal sepsis and provides insight into pathways that may be relevant to the development of diagnostic and treatment methods for sepsis.

## Supplementary Material

Supplemental Material

Supplemental Material

## Data Availability

The datasets used and/or analyzed during the current study are available from the corresponding author upon reasonable request.
